# A Swine Model of Changes in the Neuronal Electromagnetic Field After Traumatic Brain Injury: A Pilot Study

**DOI:** 10.7759/cureus.41763

**Published:** 2023-07-12

**Authors:** James Brazdzionis, Mohamed M Radwan, Finosh G Thankam, Merlin Rajesh Lal, David Baron, David A Connett, Devendra K Agrawal, Dan E Miulli

**Affiliations:** 1 Neurosurgery, Riverside University Health System Medical Center, Moreno Valley, USA; 2 Translational Research, College of Osteopathic Medicine of the Pacific, Western University of Health Sciences, Pomona, USA; 3 Psychiatry and Behavioral Sciences, College of Osteopathic Medicine of the Pacific, Western University of Health Sciences, Pomona, USA

**Keywords:** neuronal circuit, traumatic brain injury, fast fourier transform, controlled cortical impact, emf, sensors, electromagnetic field, tbi, swine model

## Abstract

Background

Traumatic brain injury (TBI) is a global cause of disability and mortality. Treatment depends on mitigation of secondary injury resulting in axonal injury, necrosis, brain dysfunction, and disruption of electrical and chemical signaling in neural circuits. To better understand TBI, translational models are required to study physiology, diagnostics, and treatments in homologous species, such as swine. Electromagnetic fields (EMFs) from altered neural circuits can be measured and historically have been reliant on expensive shielding and supercooling in magnetoencephalography. Using proprietary induction sensors, it has been found that a non-invasive, non-contact approach with an engineered Mu-metal and copper mesh-shielded helmet effectively measures EMFs. This has not yet been investigated in swine models. We wished to evaluate the efficacy of this technology to assess TBI-dependent EMF changes in swine to describe the efficacy of these sensors and this model using a gravity-dependent controlled cortical impact (CCI).

Methods

A Yucatan miniswine was evaluated using non-contact, non-invasive proprietary induction sensors with an engineered dual-layer Mu-metal and interlaced copper mesh helmet with sensors within EMF channels connected to a helmet. Swine EMF recordings were obtained prior to induced gravity-dependent CCI followed by post-TBI measurements. Behavioral changes and changes in EMF measurements were assessed. EMF measurements were evaluated with an artificial intelligence (AI) model.

Results

Differences between room “noise” EMF measurements and pre-TBI swine electromagnetic field measurements were identified. Morphological characteristics between pre-injury and post-injury measurements were noted. AI modeling differentiated pre-injury and post-injury patterns in the swine EMF. Frequently identified frequencies seen post-injury were peaks at 2.5 Hz and 6.5 Hz and a valley at 11 Hz. The AI model identified less changes in the slope and thus decreased variation of EMF measurements post-TBI between 4.5 Hz and 7 Hz.

Conclusions

For the first time, it was identified that cortical function in a swine can be appropriately measured using novel induction sensors and shielding isolated to a helmet and EMF channels. The swine model can be appropriately differentiated from the external noise signal with identifiably different pre-injury and post-injury EMFs. Patterns can be recognized within the post-injury EMF due to altered neural circuits that can be measured using these sensors continuously, non-invasively, and in real time.

## Introduction

Traumatic brain injury (TBI) affects millions of patients globally each year with management of TBI limited to prevention of the initial TBI and then preventing complications and secondary injury [1,2]. Moreover, the pathological insult and damage caused by TBI are limited due to changes in complex cellular mechanisms and poor renewal mechanisms of the brain and rely on a complex system of neuroplasticity [3]. In addition, injury to the neurons and glial cells following the incident TBI alters neuronal transmission and impairs the ability to create action potentials and perform spatial and temporal summation to produce appropriate excitatory and inhibitory post-synaptic potentials [4]. This is compounded by the pro-inflammatory milieu from the secondary injury responses with cytokine release, oxidative stress, axonal degeneration, glutamate release, and necrosis [5-7]. Such adverse responses cause brain dysfunction, impairing participation in appropriate and normal activities such as disruption in motor function, sensation, and cognitive status from abnormalities in cellular and neural circuits [8]. Therefore, treatment relies on optimizing conditions for neuroplasticity with reduction of risk factors of secondary injury requiring complex neurocritical care interventions, observation, and treatments.

TBI causes physiologic abnormalities in electrochemical signaling in the brain. Necessarily, as all electrical signals create a magnetic field, there must be resultant abnormalities within the magnetic field of neurons in the areas of injury. Interestingly, the electromagnetic field (EMF) has been found to be measured in humans through novel induction sensors in a non-contact, non-invasive method [9-14]. Also, using a shielded helmet and induction sensor construct has efficaciously removed extrinsic noise data from the external EMF and non-invasively isolated the components to the subject continuously in real time [10]. Clinically, allowing for real-time evaluation of functionality of neural circuits could allow for more targeted treatments and provide additional monitoring capabilities to neurointensive care units to promote optimal recovery. Even more so, defining the degree of injury has relied on computed tomographic studies, magnetic resonance imaging, and clinical exam which is limited to hospital-based studies. If diagnostic evaluation could be obtained in a prehospital setting and evaluate functional disruption, there may be opportunities for better utilization of resources and direction of patients to appropriate centers for care. Using helmet-based shielding technology with compact induction sensors, portability has the potential to be vastly improved for scalability in clinical use. Unfortunately, the lack of proper translationally relevant animal models in TBI hurdles understanding TBI and its effects on the EMF derived from neuronal circuits. However, interestingly, the intrinsic similarities to human physiology and biochemistry, analogous size, healing responses, and genetic homology have made swine an appropriate model to simulate and replicate TBI [15]. Even though variations in topography and anatomical organization could be in the swine brain, the close similarities in cellular, molecular, and pathophysiological responses translate the swine models to clinical arenas [16].

Moreover, multiple approaches have been utilized to emulate traumatic brain injury. These approaches include models relying on acceleration and deceleration injury and controlled cortical impact (CCI). CCI models produce a focal injury from a direct impact on the brain cortex itself [17,18]. This model relies on a standardized impact to an animal under general anesthesia, following craniotomy the cortical surface is impacted to the degree of injury. The traditional impact force in a swine CCI model is approximately 3.5 m/sec [17]. These approaches have a limitation secondary to the cost associated with the device, inability requirement of a craniotomy and lack of diffuse effects associated with TBI [19]. To address the challenge of cost, we describe an approach utilizing gravity and a steel sterilized ball to create a controlled impact and subsequent TBI at the cortical surface and brain parenchyma.

Post-impact, the cortical surface and brain parenchyma subsequently suffer an impact and an injury. Due to injury, it is known that dysfunction of the electrochemical signaling from the neuronal structures will be altered from the TBI. Therefore, the magnetic fields generated by the swine model subsequently should be affected by cortical injury. Studies evaluating these effects on the EMF have been limited but may have large-scale clinical applications as a potential diagnostic modality to evaluate TBI with its effects on neural circuits in real time. Therefore, we aimed to evaluate the efficacy of using non-invasive techniques with a helmet and induction sensor construct on a swine translational model that was previously used in human models to evaluate normal humans. In doing so, we aimed to evaluate whether this modality would be appropriate in evaluation of the baseline neural-generated EMF in swine. We secondarily aimed to evaluate whether changes in this magnetic field generated by TBI using a gravity-dependent CCI model could be successfully evaluated in a swine model.

## Materials and methods

**Figure 1:**
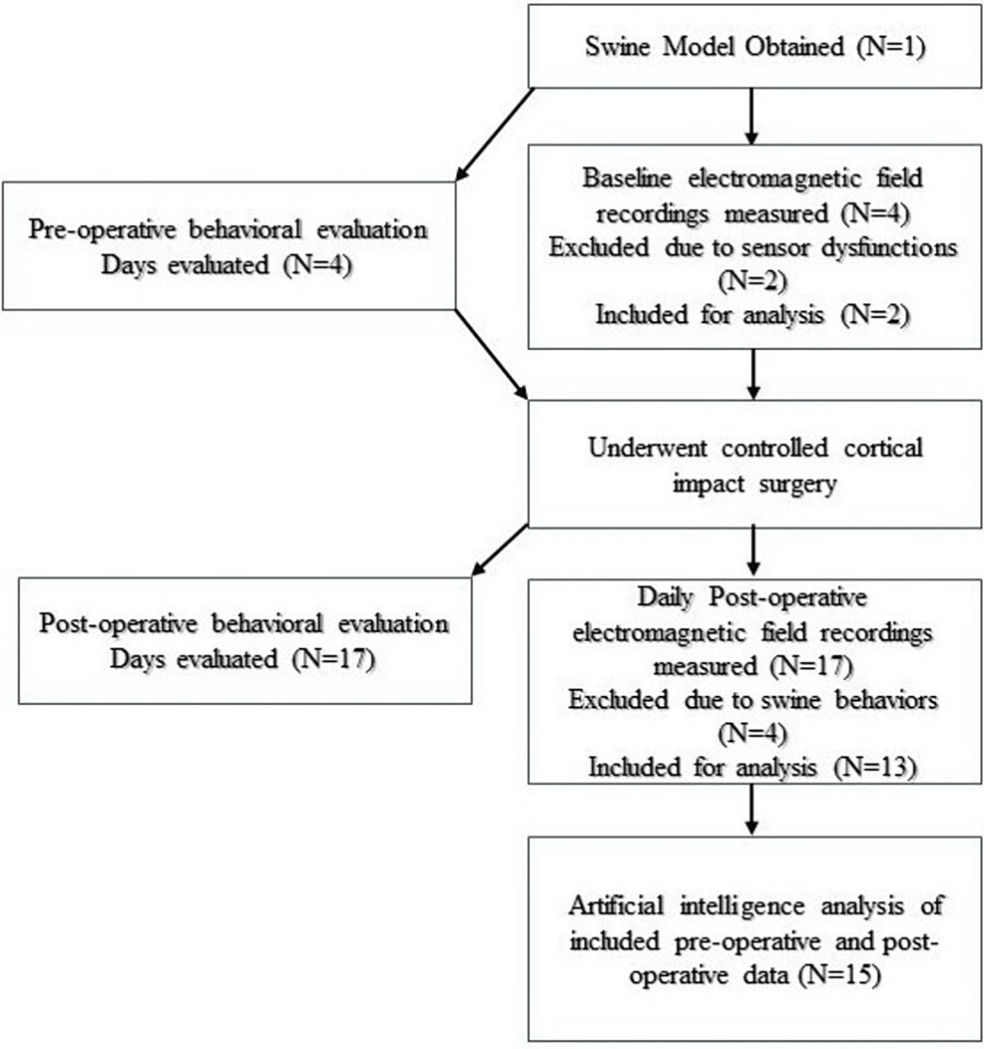
Diagram of the study design A flow chart is shown identifying the corresponding steps in the procedure that the swine model participated in

The animal protocol for this study was approved by the Western University of Health Sciences Institutional Animal Care and Use Committee (protocol # R23IACUC003). A diagram of the complete study design is presented in Figure 1. A 20-30kg male Yucatan miniswine (Premier BioSource, Ramona, CA) was utilized as the swine model. The subject was maintained with a normal diet with unlimited access to water. The swine model was acclimatized and conditioned using a helmet analogue prior to testing. Using proprietary induction sensors (BS-1000) designed by Quasar Federal Systems (San Diego, CA) and a shielded helmet with EMF channels as described by Brazdzionis et al. and Wiginton et al. baseline pre-operative EMF signals were measured from the swine model prior to induction of TBI using a CCI model [9-13]. A new helmet was manufactured with interior and exterior plastic layers with dual-layered Mu-metal (MuMETAL®, Magnetic Shield Corporation, Bensenville, IL) a 2.5 cm air gap, and inner and outer interlaced copper mesh following the method by Wiginton and Brazdzionis [9-13]. Sensors were placed within Mu-metal shielded EMF channels connected to the helmet. Data were obtained using a 16-bit National Instruments data card (National Instruments Corporation, Austin, TX) at a rate of 5-kilo samples per second after passing through a 2 kHz low pass filter with a 10x gain module. Detection sensitivity of the sensors was 1pT/rtHz at 1Hz and capture EMF signals between 1 Hz and 2 kHz within their cylindrical region of detection. EMF waves were analyzed using Igor ® Pro version 8 (WaveMetrics, Lake Oswego, OR) and transformed using a fast Fourier transform (FFT) algorithm to evaluate the frequency domain. For measurements, the helmet was held over the head of the minipig with the center front of the helmet oriented toward the tip of the snout. The helmet was held directly over without touching the head. The four sensors were labeled B319, Bx, By, and Bz and placed in EMF channels connected to the helmet with the positive ends directed toward the scalp. B319 was directed toward the left frontal region, Bz at the left parietal region, By to the right frontal region, and Bz to the right parietal region. Structurally, the tips of the sensors were located 2cm above the scalp surface within the helmet for measurements. Treats were given to the pigs to train them and used during measurements for consistent measurements. Data were recorded, and test periods containing a 20-second consecutive bin (100,000 data points per Hz) were analyzed where the helmet was ensured to be consistently over the pig’s head during the 20-second bin for analysis using the Igor software. Prior to CCI surgery, baseline EMF readings were measured daily. The swine was assessed and evaluated qualitatively to assess their normal functional activities such as eating, ambulating, and vocalization.

CCI model

A CCI model was employed to induce a TBI in the subject. To create an impact, a gravity model was utilized using 3 feet of pipe and a steel ball with a mass of 8.369 grams. It was calculated that a vertical drop through the piping with the steel ball would fall at a rate of 3.579 meters/second consistent with previous studies of impact [17]. Therefore, it was planned to perform a craniotomy to create a 1 cm cranial defect using a mechanical burr under standard sterile conditions under general anesthesia and orient the tubing vertically and perpendicular to the burr hole and drop the steel ball onto the cortical surface.

The cortical target for the CCI model was the left cruciate gyrus; the motor strip for swine with the incision guided by previous anatomical studies of swine using the bregma as a landmark [16,20].

Anesthesia utilized in this study included acepromazine, ketamine, Telazol, xylazine, propofol, and isoflurane. Vital signs in the swine were monitored throughout the surgery including pre-induction, post-induction, pre-injury, and post-injury.

Surgical procedures and measurements

After induction, the pig was placed in a stereotactic head holder to ensure the craniotomy site was oriented at the apex, and pressure points were padded with gauze. The baseline EMF was measured in the room on the operating table prior to measurement in the subject’s cranium ensuring consistent orientation described above and baseline EMF measurements were taken. The site for the craniotomy was then planned and marked. The hair at the site of the craniotomy was then removed using hair clippers. The site was sterilized using alcohol and povidone iodine. The pig was sterile draped and local anesthesia was infiltrated at the site of the incision. An approximately 3-4 cm linear incision was made and using monopolar cautery was continued to bone and the periosteum was removed using a periosteal elevator. A Hudson brace was used to drill a burr hole through the calvarium to expose the dura. Upon confirmed dural exposure, the metal guide tube for the steel ball was inserted into the burr hole and oriented vertically. The steel ball was dropped from the top of the tube. The tubing was removed, and the steel ball was identified within the burr hole site on the dura. The wound was then copiously irrigated, and hemostasis was obtained. The site of the burr hole was lined with bone wax and a layered closure was performed using absorbable suture. A skin adhesive was applied and after drying a transparent film dressing was placed.

Post-operative EMF measurements were obtained while under anesthesia. General anesthesia was then stopped, and the pig was recovered in their associated pen. The pig was allowed to recover for approximately 1 hour until ambulatory at which point it would become safe for them to take oral intake and the associated licorice utilized in measurements. Once ambulatory, a post-operative EMF measurement was obtained.

Post-operative baseline pen measurements (taken from the floor to evaluate external EMF) as well as EMF measurements of the swine model were taken daily until sacrifice on post-operative day 21. The pig was evaluated daily to ensure appropriate oral intake, ambulation, and activity. As the left cruciate gyrus was targeted, abnormalities regarding right-sided extremity and neck movements were especially assessed. No mortality was encountered during the procedures or post-operative management and the animals returned to normal cage activities in 2-3 days.

EMF analysis

Daily EMF values were assessed by two investigators (DM and JB), and the prominent peaks and valleys were recorded for sensor B319. Peaks were defined as the region of maximal amplitude where there was an inflection point prior to having an amplitude with less absolute value than that inflection point. Valleys were defined as inflection points were the amplitude had its least value as it progressed along the frequency domain prior to having an increasing value. Each peak and valley inflection point were recorded accordingly. Sensor B319 was selected for this analysis as sensor B319 was oriented such that it would be located over the induced lesion. These peaks and valleys were recorded, and overall patterns were assessed in pre-operative and post-operative values using the assistance of a large language model (LLM) AI software. On post-operative days 5, 8, 9, and 10, the swine had inconsistent behavior consistent with impulsivity and depression from the TBI and therefore, EMF measurements these days were omitted from analysis.

## Results

Animal EMF analysis

**Figure 2:**
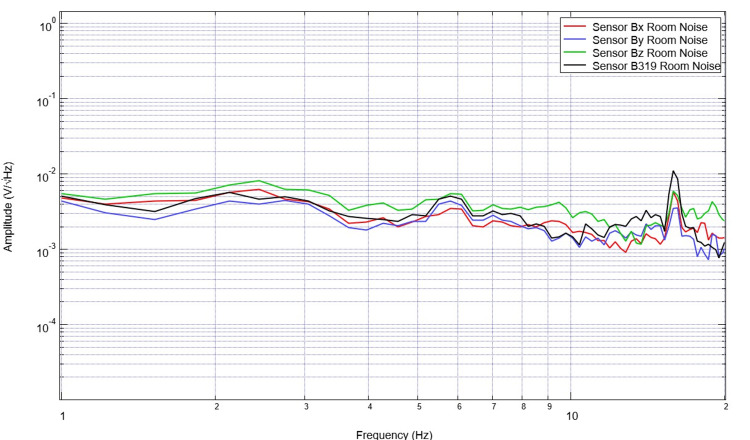
Baseline room electromagnetic field recordings are identified by the sensor Room noise obtained using the helmet without a subject is portrayed. Sensor orientation within the helmet was: B319 located left frontal, Bz located left parietal, By located right frontal, and Bz right parietal.

**Figure 3:**
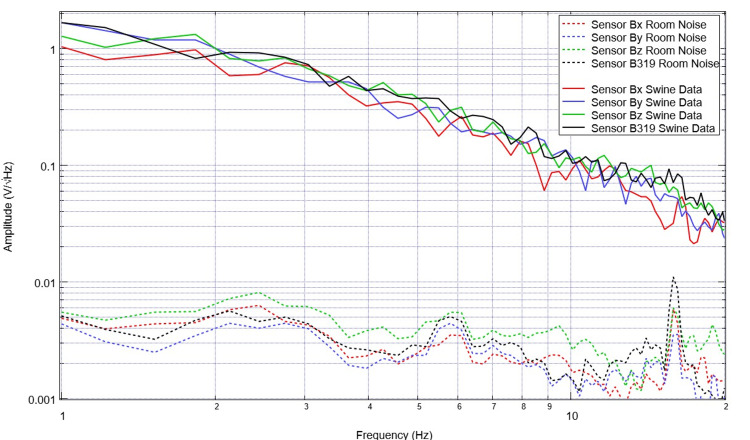
A comparison graph identifying baseline room noise data and swine pre-operative data Sensor orientation within the helmet was: B319 located left frontal, Bz located left parietal, By located right frontal, and Bz right parietal. Amplitudes and morphology are vastly different comparing swine and noise data.

Baseline EMF recordings were evaluated for each daily measurement. The range of amplitudes for the noise EMF data is much less than that of that seen in the swine model (Figure 2). Furthermore, the morphology of the waveforms is vastly different comparing the baseline room EMF compared to that generated in a swine model (Figure 3).

**Figure 4:**
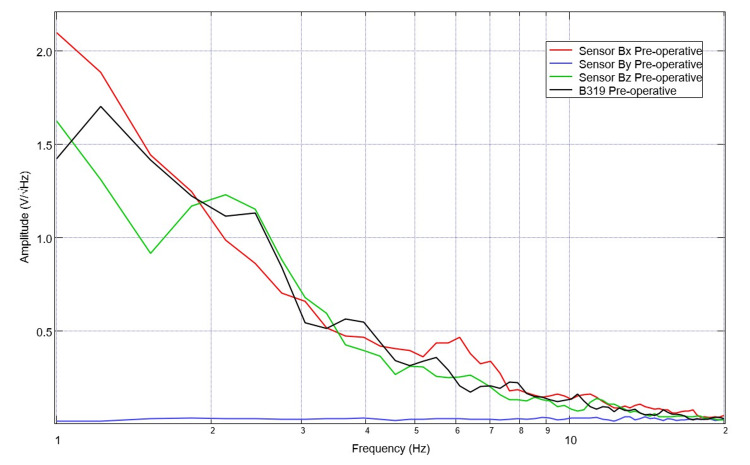
Baseline swine field recordings identified by sensor Sensor orientation within the helmet was: B319 located left frontal, Bz located left parietal, By located right frontal, and Bz right parietal. There are identified morphological characteristics in each wave including peaks defined as the amplitude where-in two adjacent points are both less than the peak, and valleys corresponding to the amplitude where both adjacent points are greater than the valley.

Waveforms recorded through the sensors are generated through an FFT transformation that demonstrates the frequency domain and the results are not portrayed in the time domain. As such, the data represent the summative potential within a given frequency over the evaluated period. Plotted data is from a 20-second bin when the helmet was identified to be centered over the pig with minimal movement of the pig. Within baseline EMF data of the pig, there are noted peaks and valleys within the graph seen in Figure 4. These peaks and valleys within this pre-operative baseline are located at 1.8, 2.6, 3.6, 4.2, 5.2, 6.2, 7.1, 9.5, and 11 for positive peaks in sensor B319 and 1.6, 2.4, 3.2, 4, 4.7, 5.5, 6.1, 7.5, and 10.5 for the inflection point of the valleys noted in the sensor B319 waveform.

Data were evaluated daily for each pre-operative day with characterization of these noted peaks and valleys for 20 second bins. Overall, there were four days of preoperative data assessed however only two were utilized for analysis due to data set issues within post-processing with signal drop-out during the initial days of testing. The artificial intelligence (AI) model was limited in assessment of pre-operative assessment due to the limited sample size of this pilot study.

**Figure 5:**
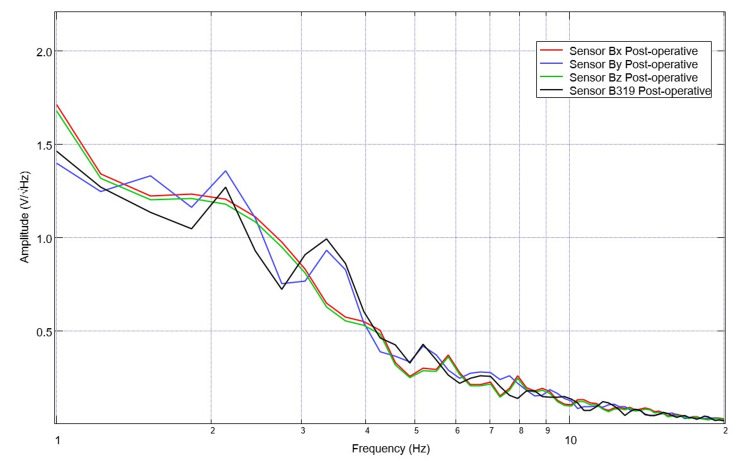
Post-operative electromagnetic field recordings identified by sensor Post-operative electromagnetic field measurements are identified for the swine model. Sensor orientation within the helmet was: B319 located left frontal, Bz located left parietal, By located right frontal, and Bz right parietal.

**Figure 6:**
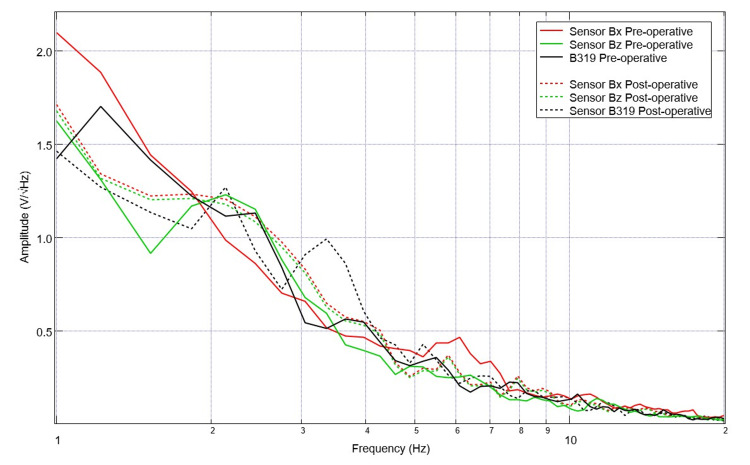
A comparison of pre-operative and post-operative swine electromagnetic field recordings identified by the sensor Sensor orientation within the helmet was: B319 located left frontal, Bz located left parietal, By located right frontal, and Bz right parietal. Morphologic differences are noted in slopes, the number of peaks and valleys and location of peaks and valleys comparing pre-operative and post-operative waveforms.

Post-procedure data were similarly investigated. A representative graph of the measured EMF is seen in Figure 5 from post-operative day 2. For sensor B319, this representative graph has identified peaks with inflection points at 1.5, 2.5, 3.5, 5.2, 6.1, 7, 8.5, 10, and 11 and valleys with associated inflection points at 2.2, 2.7, 3.1, 4.5, 5.8, 6.7, 6.5, 9.4, 10.5. Of note, during data collection there were four post-operative days where the pig would not remain still for testing, was more agitated and depressed and these days were not included in analysis (post-operative days 5, 8, 9 and 10). Overall, within the post-operative data set thirteen days of post-operative values were obtained and analyzed including post-operative day 0 values obtained post-op during recovery from anesthesia while in a pen. A comparison graph of pre-operative and post-operative data is seen below in Figure 6.

Behavioral observations

Behaviorally, initially the pig continued to eat without dysfunction post-operatively. Immediately following recover the pig appeared ataxic. On post-operative days 1 and 2, it had mild difficulties with ambulation regarding its right rear foot with a degree of continued ataxia and wide based gait. However, the pig quickly recovered, and no obvious weakness was noted and started active cage activities on post-operative day 3. Interestingly, upon recovery of its weakness, the pig was found to be impulsive with increased activity post-operatively with increased jumping and climbing on the walls within the pen especially at times of feeding. Of note on post-operative days 5, 8, 9, and 10, the pig was demonstrating signs of depression consistent with findings in swine TBI where-in it was more agitated and refused measurements. During these days, the pig would not take licorice from the examiner noting decreased appetite. Consistent with previous studies, an increase in blood pressure was noted post-impact with an increase in blood pressure from 98/30 mm/Hg just prior to induced TBI to 112/44 mm/Hg after impact with the steel ball during craniotomy [17].

AI analysis

As the pre-operative data set consisted of two days of usable data, an analysis of patterns was limited when isolated to just the pre-operative data set. With further analysis, using the AI model the similarities within the post-operative data set were that there were repeated values within the peaks and valleys. The most frequent of which within the valleys this was identified to be the value of 11 Hz where it was identified 7 times within the 12 evaluated days. Within the valleys post-operatively it was noted that overall, between frequencies of 4.5 and 7 Hz there were not as many identified inflection points as prior to 4.5 Hz and after 7.5 Hz. This identifies that there were less fluctuations without changes in directionality of the slope of the graph with fewer peaks and valleys between these points. The AI model identified within the inflection points of the valleys that the overall range expanded over time such that the range of observed valleys was greater post-operatively. Pre-operatively there was a relatively smoother progression of valleys pre-operatively compared to post-operatively with more variation in the spacing between valleys.

Within the peaks (positive inflection points), the AI model identified several repeated frequencies within the post-operative data. When using a 50% threshold of frequencies seen in multiple data sets, the values of 2.5 Hz (repeated six times) and 6.5 Hz (repeated seven times) displayed repetition.

Moreover, the AI model also identified that the pre-operative data set contained a fixed sample size of nine different peaks while the post-operative set ranged between 6 and 11 peaks. The AI model also identified that despite the several noted repeated peaks post-operatively the data set has an overall larger amount of variation of the number of peaks, values of peaks and overall pattern of peaks within the post-operative data set compared to the pre-operative data set. Thus, note that the overall variation in patterns seen day to day was much more variable post-operatively compared to pre-operatively. It further identified that these two data sets were distinct with distinct patterns.

## Discussion

TBI is common and from clinical and socioeconomic perspectives is associated with high costs, morbidity, and mortality, being incident to nearly 1/3 of traumatic deaths in the United States [21]. Additionally, healing mechanisms within brain tissue are complex, and the lack of translationally relevant large animal models offers additional challenges. Hence, research on TBI is critical for improved outcomes and further understanding of the disease process gearing effective treatments. Furthermore, appropriate models of TBI are important for designing novel treatments for better management of TBI. We have successfully developed the CCI swine model of TBI and effectively utilized induction sensors with a magnetically shielded helmet and EMF channels to detect brain activity pre- and post-injury non-invasively.

Assessing behavioral changes within our swine model identified that the subject became more impulsive, declined treats for several days, and demonstrated weakness immediately post-operatively. Previous studies evaluating swine identified hyperactivity and gait dysfunction similar to what was observed in this pilot [22].

EMFs generated by cortical neurons in their physiologic signaling mechanisms have effectively been measured using induction sensors with Mu-metal shielded helmets and EMF channels. To the best of our knowledge, this is the first attempt to optimize EMF measurements in a translationally worthwhile swine model. Alternative methods of measurement of cortical functioning including electroencephalography (EEG) and magnetoencephalography (MEG) have been clinically employed [23,24]. Importantly, induction sensors and specific shielding technology used in our study allow continuous, non-invasive, real-time examination without the need for supercooling or expensive superconducting quantum interference devices used in MEG lowering cost. The devices used in this study are portable and have simplicity of application.

Our findings revealed considerable differences between baseline noise activity that may be seen deep into the sensors beyond the open end of the helmet and that which is generated by the pig. For the first time, this establishes the efficacy of this model in the assessment of the generated EMF due to the substantial differences noted in amplitude and morphology within the frequency domains. It is important to note that amplitude has a denominator of the square root of Hertz, and therefore as a necessary effect, there are noted decreases in amplitude along the x-axis. Additionally, the efficacy of these measurements establishes that the sensors effectively evaluate the generated EMF by the swine model both pre-operatively and post-operatively which distinguishes the neuronal circuit-driven EMF from the in-room EMF noise. Furthermore, as pre-operative data were easily assessed, it identifies the efficacy of this technology to measure the EMF generated by cortical neuronal and cellular circuits through an intact skull and scalp, continuously, non-invasively in real time through a portable helmet-based system.

AI was utilized in assessment of data, and the AI system effectively identified that pre-operative and post-operative data sets were different in pattern. Other highlighted AI observations included that post-operatively there were fewer changes in slope in the waveform between 4.5 and 7.5 Hz leading. Despite this, the AI identified more variation in the post-operative peaks compared to the pre-operative baseline EMF measurements. These changes may represent changes in firing due to lesioning effect and therefore changes in neuronal signaling and neuronal circuits identified by the sensors.

Important valleys identified by the AI were at 11 Hz frequency post-operatively. Additionally, dominant peaks were found at 2.5 Hz and 6.5 Hz post-operatively. These values may represent dominant frequencies that are important in signaling as the Fourier transformed data represents an increased frequency of observations of a certain frequency as measured by amplitude. Therefore, areas with more summative data are more frequently encountered frequencies and may be areas for further investigation with regard to spatial and summation for dominant signaling and pathways.

Effects of TBI or other neurological-based lesions have not yet been demonstrated using this induction sensor, shielded helmet, and EMF channel construct in previous studies as all previous studies used healthy human models [9-13]. Therefore, it is noted that these differences are secondary to the TBI sustained in the swine model due to alterations and injury in the cortical tissue. This injured cortical tissue necessarily causes alterations in neuronal signaling as the impacted neurons and glial cells fail to participate in normal functions post-injury as noted through TBI physiology [8]. Therefore, this induced CCI model is an appropriate and feasible model of TBI with noted changes in the patterns of activity pre-operatively and post-operatively. This model also was reliable and consistent with other CCI models with increases in blood pressure post-injury [17]. Importantly, pre-operative and post-operative values differed, and therefore the neuronal circuits were appropriately altered, and this model is translationally relevant for TBI. Finally, due to these findings, this sensor and helmet system with EMF channels is an efficacious way to continuously and non-invasively evaluate neuronal activity and circuits through an intact scalp and skull as obvious changes were seen comparing pre-operative and post-operative measurements.

The findings from this study are translatable to human models as these devices may be utilized as diagnostic devices to evaluate TBI and potentially other neuronal injuries within human subjects. Further studies are warranted to design and develop a larger database of pre-operative EMF waves amongst multiple subjects and post-operative EMF waves with similar injuries for generating functional maps with the assistance of unsupervised learning with artificial intelligence modeling. Furthermore, other future directions for investigation may include guided stimulation of neuronal EMF from circuits. Transcranial magnetic stimulation has recently undergone research on its effects on TBI with histological and behavior improvements in rodent models post TBI [25,26]. As this investigation measured cortically generated EMF with differentiation of pre-operative and post-operative states, further studies geared at EMF stimulation at abnormal frequencies measured post-operatively with a targeted treatment with stimulation at abnormal frequencies are clinically promising in TBI.

Limitations

This study has several limitations. This was conducted as a pilot study with a small sample size and relatively few measurements pre-operatively which limit the certainty in the pre-operative baseline that will need to be assessed for reproducibility in larger trials. The usage of LLM AI for assessment can be prone to hallucination effects. Further studies are warranted to confirm the findings using additional computer programs and AI with the use of unsupervised learning algorithms and random forest model analysis. Nonetheless, the findings from this study offer promise in designing translational approaches for the management of TBI and for discerning changes in neuronal circuitry non-invasively and in real time.

## Conclusions

Evaluation of the cortical function in a swine can be completed through usage of portable electromagnetic field measuring induction sensors and appropriate shielding with a magnetically shielded helmet continuously in real-time. For the first time, with this technology, it was noted that there are differing patterns noted by the induction sensors comparing baseline room activity without the swine subject within the helmet and with the swine subject. It was noted by AI modeling systems that induced TBI using a CCI model alters patterns in neuronal activity measured through EMF and therefore neuronal circuits. Therefore, this translational model and technology are efficacious in modeling and evaluating TBI. These changes in neuronal circuits can be effectively measured by this sensor and portable helmet system in a non-invasive fashion and were observed to be different comparing pre-operative and post-operative measurements in a Yucatan miniswine model for TBI.
